# Autologous hematopoietic stem cell transplantation in relapsing-remitting multiple sclerosis: comparison with secondary progressive multiple sclerosis

**DOI:** 10.1007/s10072-017-2933-6

**Published:** 2017-04-10

**Authors:** Bonaventura Casanova, Isidro Jarque, Francisco Gascón, Juan Carlos Hernández-Boluda, Francisco Pérez-Miralles, Javier de la Rubia, Carmen Alcalá, Jaime Sanz, Javier Mallada, Angeles Cervelló, Arantxa Navarré, María Carcelén-Gadea, Isabel Boscá, Sara Gil-Perotin, Carlos Solano, Miguel Angel Sanz, Francisco Coret

**Affiliations:** 1 Neuroimmunology Unit, Hospital Universitari I Politècnic La Fe, Avd Abril Martorell, 46026 Valencia, Spain; 2 Hematology Service, Hospital Universitari I Politècnic La Fe, Valencia, Spain; 3 Neuroimmunology Unit, Hospital Clínic Universitari de València, Valencia, Spain; 4 Hematology Service, Hospital Clínic Universitari, Valencia, Spain; 5 Hematology Service, Hospital Universitari Dr. Peset and Universidad Católica “San Vicente Mártir”, Valencia, Spain; 6 Neurology Service, Hospital General de Elda, Elda, Spain; 7 Neurology Service, Hospital General Universitari de València, Valencia, Spain; 8 Neurology Unit, Hospital de Sagunt, Valencia, Spain

**Keywords:** Autologous hematopoietic stem cell transplantation, Multiple sclerosis, Secondary progressive multiple sclerosis, Immunotherapy, Immunosupression, Neurodegeneration

## Abstract

The main objective of our work is to describe the long-term results of myeloablative autologous hematopoietic stem cell transplant (AHSCT) in multiple sclerosis patients. Patients that failed to conventional therapies for multiple sclerosis (MS) underwent an approved protocol for AHSCT, which consisted of peripheral blood stem cell mobilization with cyclophosphamide and granulocyte colony-stimulating factor (G-CSF), followed by a conditioning regimen of BCNU, Etoposide, Ara-C, Melphalan IV, plus Rabbit Thymoglobulin. Thirty-eight MS patients have been transplanted since 1999. Thirty-one patients have been followed for more than 2 years (mean 8.4 years). There were 22 relapsing-remitting multiple sclerosis (RRMS) patients and 9 secondary progressive multiple sclerosis (SPMS) patients. No death related to AHSCT. A total of 10 patients (32.3%) had at least one relapse during post-AHSCT evolution, 6 patients in the RRMS group (27.2%) and 4 in the SPMS group (44.4%). After AHSCT, 7 patients (22.6%) experienced progression of disability, all within SP form. By contrast, no patients with RRMS experienced worsening of disability after a median follow-up of 5.4 years, 60% of them showed a sustained reduction in disability (SRD), defined as the improvement of 1.0 point in the expanded disability status scale (EDSS) sustains for 6 months (0.5 in cases of EDSS ≥ 5.5). The only clinical variable that predicted a poor response to AHSCT was a high EDSS in the year before transplant. AHSCT using the BEAM-ATG scheme is safe and efficacious to control the aggressive forms of RRMS.

## Introduction

Multiple sclerosis (MS) is a heterogeneous inflammatory disease affecting the central nervous system (CNS), in which a deep immunological alteration is in the origin of the pathologic phenomena that occurs in MS patients and lead to the progressive and irreversible degeneration of the CNS [1].

Despite advances in the treatment of MS, many patients do not respond to available drugs and require other therapeutic strategies to control disease activity and to prevent the progression of disability [2, 3].

Autologous hematopoietic stem cell transplantation (AHSCT) has been recognized since the final of the last century as a therapeutic option for very aggressive MS patients [4]. Initially, patients with high disability and progressive MS forms were included in the series of cases studied, but promptly, this strategy was abandoned because of significant mortality without a clear effect on the disease progression [5, 6]. However, MS patients with high inflammatory activity in the earliest stages of the disease, who show refractoriness to standard therapies, may respond well to AHSCT, with a transplant-related mortality that has decreased from 5.3% [7] to 1.3% [8].

Although the benefit to treat MS has not been demonstrated in randomized clinical trials, the cumulative clinical experience summarized in several meta-analysis has shown a progression-free survival at 5 years of nearly 80% in aggressive MS patients [9]. Notably, the vast majority of patients were transplanted when they were in the progressive stage of the disease. According to recent data published in the largest observational study, the results of AHSCT in relapsing-remitting multiple sclerosis (RRMS) (16% of the series) progression-free patients ranged from 90% [10] to 100% [11].

We report here our experience in the real life in patients with MS who underwent AHSCT in a 16-year period, with special attention to the long-term evolution of RRMS and the differential response compared to SPMS treated with AHSCT.

## Methods

### Eligibility criteria

The local Ethics Committee of Clinical Investigation of the University Hospital La Fe and University Clinic Hospital of València approved the treatment protocol. Each patient was selected at the Neuroimmunology Units of the participant Centers and signed the informed consent. The AHSCT procedure was carried out in the Transplant Unit of the Hematology Department.

We selected patients according to the following criteria: RRMS or SPMS patients under treatment with one of the MS-approved drugs for more than 1 year, who experienced one or more relapses in the previous year and worsening of at least 1 point in disability (EDSS). Overall, a total of 38 MS patients transplanted between 1999 and 2015 were included in the study, for safety results, and 31 patients followed by more than 2 years have been selected to analyzed efficacy.

### Objectives and definitions

The main objective of our work is to describe the toxicity and the long-term efficacy of AHSCT. Efficacy was assessed in patients followed for at least 24 months.

Efficacy has been measured according to four parameters: time to first relapse, time to 1 point increase in disability sustained for 6 months (0.5 if baseline expanded disability status scale (EDSS) was higher than 5.0), NEDA (no evidence of disease activity), and sustained recovery of disability (SRD). NEDA was defined as the absence of relapses and/or increases of disability according to the previous definition and no new T2 lesions or gadolinium-enhanced lesion in the MRI performed in the last control. Sustained recovery in disability (SRD) was defined as the improvement of at least 1.0 point in the EDSS, sustained for 6-months. Toxicity has been recorded according to the WHO (World Health Organization) classification, and fever due to ATG and engraftment syndrome has also been recorded.

### Stem cell mobilization and transplant procedure

Peripheral blood stem cells (PBSCs) were mobilized with cyclophosphamide (CY) 4 g/m^2^ in 1 day followed by daily granulocyte colony-stimulating factor (G-CSF) (filgrastim) 5 μg/kg subcutaneously starting from day 2 from CY until stem cell collection. Prednisone (5 mg/kg) was added to prevent cytokine release during CY administration. The target number of harvested CD34+ cells was 3 × 10^6^ to 8 × 10^6^/kg. Unmanipulated PBSCs were cryopreserved using standard techniques; when possible, a back-up dose was also stored. Conditioning regimen was carried out within 30 to 40 days after mobilization and consisted on: BCNU 300 mg/m^2^ at day 7, cytosine arabinoside 200 mg/m^2^ and etoposide 200 mg/m^2^ from day 6 to day 3, and melphalan 140 mg/m^2^ at day 2 (BEAM regimen). On day 0, cells were thawed and infused. Rabbit ATG (Thymoglobulin; Genzyme, Cambridge, MA) (3.75 mg/kg/day) was administered at days 1 and 2 for in vivo T depletion, together with prednisone (5 mg/kg). G-CSF was not administered after transplantation unless the neutrophil count was below 0.5 10^9^/L on day 12, to avoid the risk of neurologic adverse effects due to G-CSF and minimize the risk of engraftment syndrome. Standard guidelines were followed for infection prophylaxis. In particular, acyclovir (250 mg/m^2^ three times per day) was given intravenously from day 6 until hospital discharge and then orally (400 mg twice a day) for 3 months after transplantation. Trimethoprim-sulfamethoxazole was given for 3 months.

### Statistical analysis

A multivariate Cox regression analysis to predict the increase in disability has been done by two models, due to existence of collinearity between baseline EDSS and EDSS in the previous year. The first model includes gender, age at AHSCT, number of relapses in the previous 2 years, MS duration to AHSCT, and baseline EDSS, and the second model includes the same variables but baseline EDSS was replaced by the EDSS in the year before.

## Results

### Patient characteristics

From February 1999 to November 2015, 38 MS patients received high-dose chemotherapy followed by autologous stem cell infusion in two different institutions (32 at the Hospital Universitari La Fe and 6 at the Hospital Clínic Universitari of Valencia) and were included in the study. The clinical characteristics of these patients are shown in Table 1. Briefly, 28 patients were diagnosed with RRMS and 10 with SPMS. There were no demographic differences between groups, but the EDSS in the previous 2 years, the year before, and baseline was high in the SPMS, and the number of relapses was significant higher in RRMS.

**Table 1:** Clinical and demographical characteristics of patients treated with high-dose immunoablative suppression followed by AHSCT

	RRMS (*n* = 28)	SPMS (*n* = 10)	Total series (*n* = 38)
Sex (*n*, % females)	20 (71.4)	7 (70.0)	27 (71.1)
Age at AHSCT (mean, SD)	36.4 (9.1)	37.5 (9.1)	36.7 (9.1)
MS duration at AHSCT (mean, SD)	10.0 (8.5)	8.0 (4.4)	9.5 (7.6)
Relapses at year 2 before AHSCT (mean, SD)	0.89 (0.9)	0.8 (1.1)	0.8 (0.9)
Relapses in the year previous AHSCT (mean, SD)^1^	1.6 (0.9)	1.3 (1.7)	1.5 (1.1)
EDSS 2 years before AHCST (mean, SD)^2^	3.2 (1.2)	5.1 (1.1)	3.7 (1.4)
EDSS 1 year before AHSCT (mean, SD)^3^	3.8 (1.1)	5.4 (0.8)	4.3 (1.3)
Baseline EDSS (mean, SD)^4^	5.0 (1.3)	6.0 (0.7)	5.3 (1.2)
MRI			
Number of Gd-enhanced lesions (mean, SD)	2.2 (4.4)	1.3 (2.3)	2.0 (3.9)
Patients with Gd-enhanced lesions (*n*, %)	17 (60.7)	3 (33.3)	20 (54.1)
Cerebrospinal fluid			
Presence of oligoclonal G bands (*n* = 30) (*n*, %)	21 (91.3)	7 (100)	28 (93.3)
Presence of oligoclonal M bands (*n* = 25) (*n*, %)	9 (42.9)	2 (50.0)	11 (44.0)
Previous treatments, no. (%)			
One treatment	4 (14.2)	2 (20.0)	6 (15.7)
Two treatments	7 (25.0)	4 (40.0)	11 (28.9)
Three treatments	8 (28.5)	1 (10.0)	9 (23.8)
Four treatments	3 (10.7)	3 (30.0)	6 (15.7)
Five treatments	3 (10.7)	–	3 (7.8)
Six treatments	3 (10.7)	–	3 (7.8)
Patients that received immunosuppressors	24 (85.7)	8 (80.0)	32 (84.2)
Median number and range of treatments received	3 (1–6)	2 (1–4)	3 (1–6)
Mean time on treatment previous AHSCT (SD), years	4.9 (3.6)	3.5 (2.5)	4.5 (3.4)

^1^ *p* = 0.04; ^2^ *p* = 0.001; ^3^ *p* = 0.001; ^4^ *p* = 0.02

### AHSCT toxicity

Immediate transplant-related toxicity is shown in Table 2. There were no transplant-related deaths. One patient died 13 years after transplant from aspiration pneumonia after progressing and reaching an EDSS of 9.5.

**Table 2:** Toxicity related to AHSCT

	Number (%)	WHO toxicity grade			
		1	2	3	4
Gut toxicity	24 (63.1)	10	12	2	0
Skin toxicity	18 (47.3)	11	5	2	0
Mucositis	17 (44.7)	11	6	0	0
Hepatic toxicity	15 (39.4)	7	4	0	4
Others	8 (21.0)	6	2	0	0
Neurologic toxicity	5 (13.1)	4	1	0	0
Coagulation toxicity	4 (10.5)	3	1	0	0
Urologic toxicity	2 (5.2)	1	1	0	0
Lung toxicity	2 (5.2)	1	1	0	0
Endocrine toxicity	2 (5.2)	1	1	0	0
Cardiac rhythm toxicity	1 (2.6)	1	0	0	0
Cardiac function toxicity	0	0	0	0	0
Renal toxicity	0	0	0	0	0
WHO secondary adverse events	98	56	34	4	4

The more frequent side effect was gut toxicity (63.1%), followed by skin toxicity (47.3%). Only four patients scored for grade 4 toxicity according to the WHO classification (10.5%), and all four corresponded to liver toxicity. Twenty-one patients experienced fever after ATG-infusion (55.2%), and 21 patients had engraftment syndrome (55.2%).

In the long-term follow-up, three solid tumors were diagnosed: two breast carcinomas and one cervical intraepithelial neoplasm grade 2. Median time from the transplantation to the diagnosis of the solid malignancy was 5.1 years.

### Efficacy and outcome

Thirty-one patients have been followed for more than 2 years after AHSCT. Median follow-up of patients alive was 8.4 years (range 2–16), 5.9 years for RRMS patients, and 9.6 years for SPMS patients (Table 3).

**Table 3:** Individual clinical and demographical characteristics of MS patients followed by a minimum of 2 years after AHSCT

Patient and year of AHSCT	Gender	Age at AHSCT	MS duration (year)	Relapses before AHSCT		Baseline EDSS	Evolution time after AHSCT (year)	Current EDSS	Previous therapy
				2 years	1 year				
Relapsing-remitting MS patients (*n* = 22)									
#1, 1999	M	44	13.1	2	1	6.50	16.83	6.0	IFN
#2, 2004	F	37	13.4	2	1	4.00	10.83	3.5	IFN, MTX,
#3, 2005	F	30	6.4	1	3	5.00	10.75	2.0	IFN, GA, AZA, MTX
#4, 2005	F	36	3.1	1	2	5.00	10.08	4.0	IFN
#5, 2007	M	27	7.6	1	1	3.00	7.92	2.5	IFN, MTX
#6, 2008	F	33	11.9	0	1	6.00	7.67	5.5	GA, IFN, MTX, IFN
#7, 2008	F	26	9.4	2	5	5.50	7.08	2.0	IFN, MTX, IFN, NTZ, GA
#8, 2009	F	43	11.9	1	2	5.00	6.92	4.0	IFN, Cy
#9, 2009	F	44	9.0	0	1	5.50	6.75	5.5	IFN, MTX
#10, 2009	F	32	11.4	1	2	3.00	6.58	3.0	IFN, GA, NTZ
#11, 2010	M	47	7.3	0	3	4.50	5.42	3.5	IFN, GA, NTZ, Cy
#12, 2010	F	45	13.1	2	1	4.50	5.42	3.5	IFN, AZA, DCZ, Cy, GA, IFN
#13, 2011	F	30	6.1	0	1	4.00	4.92	3.5	IFN, MTX, NTZ
#14, 2012	M	12	2.8	2	2	6.00	3.83	1.0	IFN, GA, Cy
#15, 2012	F	46	7.5	2	2	3.50	3.33	3.5	IFN, NTZ, FGM
#16, 2013	F	34	5.1	0	1	5.50	2.58	4.0	IFN, NTZ, RTX
#17, 2013	M	37	13.2	1	1	4.00	2.58	4.0	IFN, MTX, IFN, NTZ
#18, 2013	M	35	9.9	1	1	6.50	2.42	3.5	IFN, NTZ
#19, 2013	F	41	7.3	0	1	4.00	2.17	2.5	Cy, IFN
#20, 2013	F	20	1.2	2	2	6.00	2.08	3.5	IFN, NTZ
#21, 2013	F	39	9.6	0	3	6.00	2.00	4.0	IFN, NTZ, FGM
#22, 2013	F	33	6.2	0	1	9.00	2.00	1.5	IFN, NTZ
Mean (SD)^a^	72.7%	35.0 (8.7)	8.4 (3.6)	1.0 (0.8)	1.7 (1.0)	5.0 (1.3)	5.9 (3.7)	3.4 (1.2)	RRMS
Secondary progressive MS patients (*n* = 9)									
#23, 2001	F	26	4.64	3	6	6.50	13.50	10.00	IFN, AZA
#24, 2002	F	42	12.12	0	1	4.50	13.17	5.50	IFN
#25, 2004	F	42	3.76	0	1	6.50	11.67	8.00	IFN, MTX
#26, 2005	F	32	7.98	0	0	6.50	10.67	8.00	IFN, AZA, MTX
#27, 2005	F	45	8.60	1	1	6.50	10.25	6.50	IFN, MTX Cy, AZA
#28, 2007	M	47	14.48	0	1	6.00	8.25	6.50	IFN, AZA, MTX, IFN
#29, 2008	F	34	15.09	0	1	6.50	7.83	8.50	IFN, MTX
#30, 2008	M	34	5.25	0	1	6.00	7.00	8.00	IFN, MTX
#31, 2011	M	22	2.51	2	0	6.00	4.08	4.00	IFN
Mean (SD)^a^	66.7%	36.0 (8.6)	8.2 (4.6)	0.6 (1.1)	1.3 (1.8)	6.1 (0.6)	9.6 (3.0)	7.2 (1.7)	SPMS
Total series (*n* = 31)									
Mean (SD)^a^	71.0%	35.7 (3.8)	8.4 (3.8)	0.9 (0.9)	1.6 (1.2)	5.3 (1.2)	8.4 (3.8)	4.5 (2.2)	

*IFN* interferon beta, *MTX* mitoxantrone, *GA* glatiramer acetate, *AZA* azathioprine, *NTZ* natalizumab, *Cy* ciclosphosphamide, *DCZ* daclizumab, *FGM* fingolimod, *RTX* rituximab

^a^Mean and standard deviation except column of gender that represents the percentage of females

**Fig. 1:**
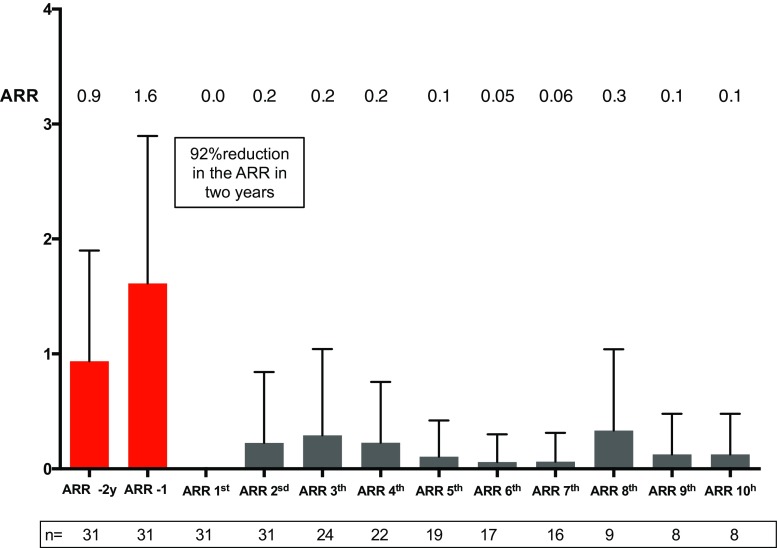
Annualized relapses rate (*ARR*) in the 2 years before AHSCT and in the following 10 years

**Fig. 2:**
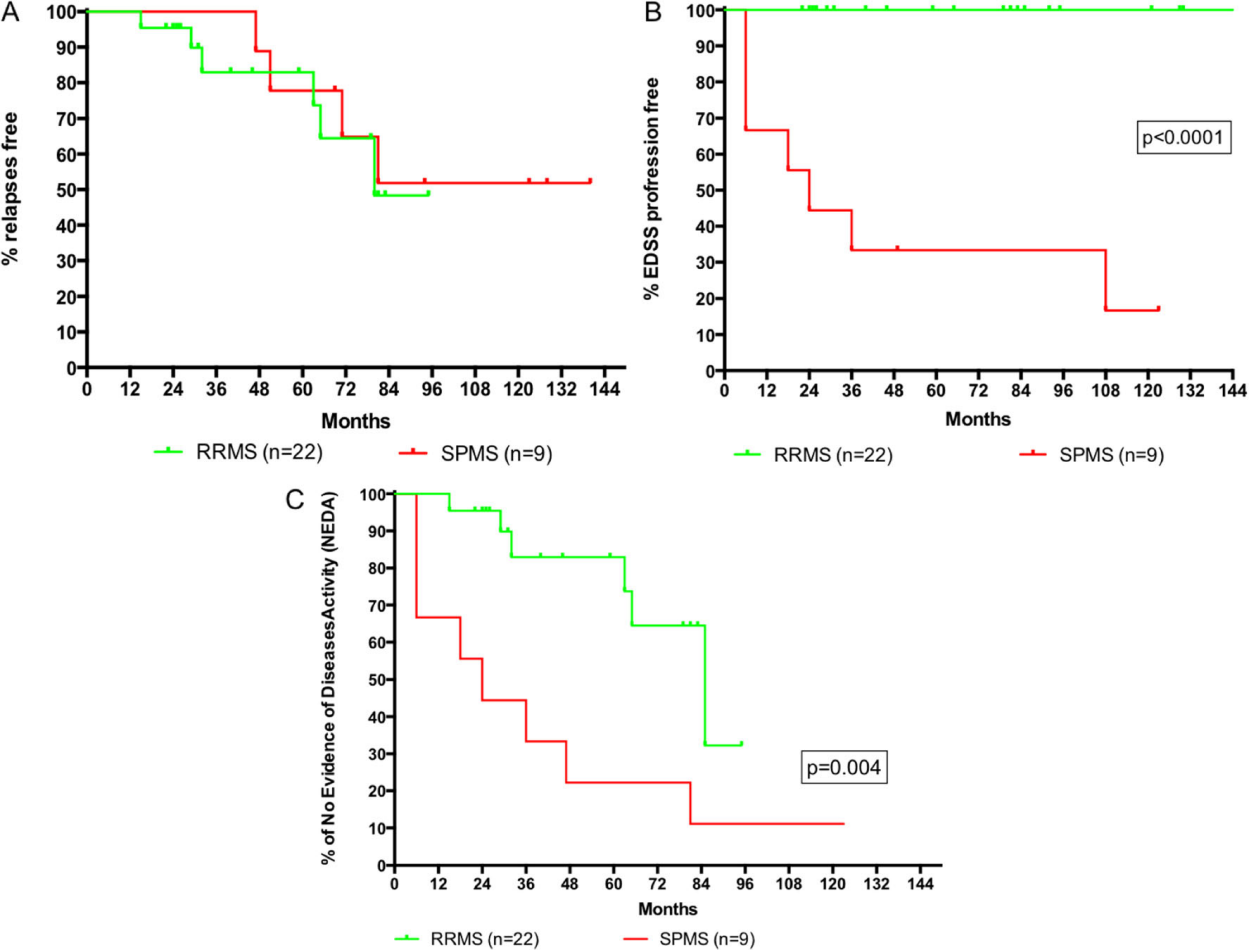
Kaplan-Meier survival analysis of the time to present: a relapse (**a**), progression of disability (**b**), and event-free -NEDA- (**c**), after AHSCT. Patients have been stratified according to the MS clinical form

The annualized relapse rate (ARR) dropped to 0 in the first year, 0.22 in the second year, then remained stable at this rate until year 5, and then fell to 0.05 in years 6 and 7 (50% of patients reached 7 years of follow-up after AHSCT). A reduction of 92% in the ARR 2 years after AHSCT was observed by comparing the ARR in the previous 2 years pre-AHSCT (2.4) to that in the 2 years post-AHSCT (0.22) (Fig. 1). A total of 10 patients (32.3%) had at least one relapse during post-transplant evolution, 6 patients in the RRMS group (27.2%) and 4 in the SPMS group (44.4%), with no differences between groups (Fig. 2a).

**Fig. 3:**
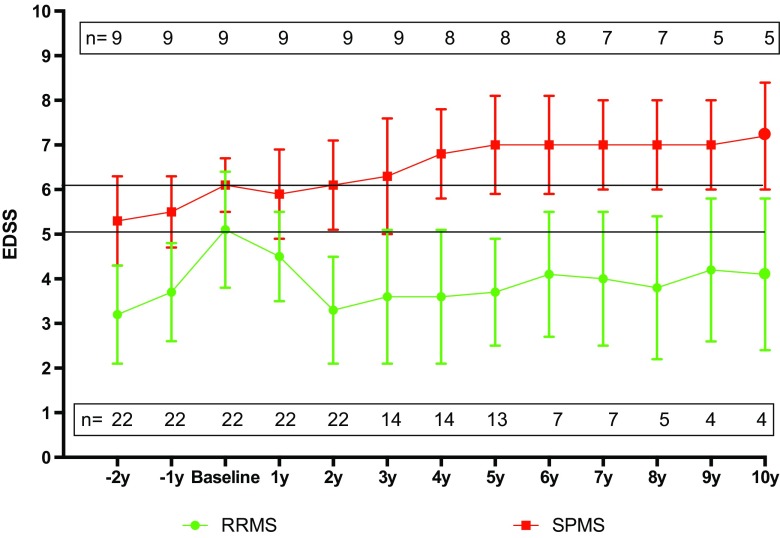
Evolution of the EDSS since 2 years before AHSCT until the 10 years after AHSCT. The patients have been stratified according to the clinical form

All patients had an increase in EDSS over 2 years prior to AHSCT (as required by inclusion criteria). After the transplant, RRMS patients showed a sustained improvement in the EDSS, while patients with SPMS remained stable the first year and then continued to progress (Fig. 3). Seven patients (22.6%) experienced progression of disability, all within SP form (non in the RRMS group) (Figs. 2b and 3).

When analyzing NEDA, some type of activity was observed in 14 patients (45.2%), 6 RRMS patients (27.3%) that relapsed and 8 SPMS patients (88.9%) with relapses and/or progression (Fig. 2c). The first MRI after AHSCT was performed at a median time of 7 months, and none showed new lesions on T2 or gadolinium-enhanced lesions. The last MRI was performed after a median time of 5 years, and in only two cases, an increase in T2 lesions was observed (both patients had suffered relapses).

Sustained recovery of disability defined as the improvement of 1.0 for 6 months was reached in 60% of RRMS patients for 7 years after AHSCT, and the remaining 40% continued stable with no worsening of disability (Table 4).

**Table 4:** Disability outcomes through year 7 after AHSCT

	Relapsing-remitting MS patients (%)	Secondary progressive MS patients (%)
Proportion of patients with 6-month sustained accumulated disability	0	78
Proportion of patients free from 6-month disability progression	100	22
Proportion of patients achieving 6-month sustained disability recovery	60	10

### Analysis of prognostic factors

A multivariate Cox regression analysis to predict the increase of disability was performed. Due to collinearity of the EDSS, two models were studied. In the second model, the EDSS 1 year prior to AHSCT increased in fivefold the probability of progression after AHSCT (Table 5).

**Table 5:** Multivariate Cox regression analysis over the risk to worsen the disability after an AHSCT

	Model 1				Model 2			
	OR	CI 95%		*p*	OR	CI 95%		*p*
		Low	High			Low	High	
Age at AHSCT	0.7	.8	1.1	ns	1.0	.8	1.1	ns
Gender	0.9	.1	5.3	ns	0.1	.0	2.1	ns
ARR in the 2 years pre-AHSCT	0.3	.4	1.3	ns	1.1	.6	2.2	ns
MS duration	1.0	.8	1.3	ns	1.0	.8	1.2	ns
Baseline EDSS	1.5	.8	2.7	ns	–	–	–	–
EDSS in the year before AHSCT	–	–	–	–	4.9	1.1	21.0	.02

Because of the presence of activity in 14 patients, 11 received treatment after AHSCT, 6 patients with RRMS who had relapses and 5 patients with SPMS. Of 6 patients with RRMS treated, 2 have been treated with rituximab and 4 with glatiramer acetate (Copaxone®); while five SPMS-treated patients, 2 were treated with rituximab and 3 with Copaxone®. So, 22 patients with RRMS (78.5%) remain untreated after 6 years of being treated with AHSCT versus 44% of patients with SPMS.

Because no patient with RRMS progressed, only the univariate Cox regression analysis was done for the variable clinical course, being the HR 21.95 (CI 95% 2.6–177.0).

## Discussion

AHSCT is an alternative therapy to manage aggressive MS patients. Definition of “aggressive MS” was based on the number of relapses and the increasing of disability while patients are under disease modifying treatment. Using these criteria, 38 patients have been studied and 31 followed for more than 2 years after AHSCT: 22 were diagnosed with a RRMS form and 9 with a SPMS. Main results in terms of efficacy were the dramatic reduction in relapses and ARR in the two clinical forms, and the arrest in the increasing of disability in RRMS, with 60% of patients improving their disability by 1.0 point or more, observing a 6-month sustained reduction in disability after a mean follow-up of 6 years [12–14].

Since the first descriptions of AHSCT in MS [15], several protocols for mobilization and conditioning have been used, with or without ex vivo CD34+ selection, and anti-thymocyte globulin as an in vivo T cell depletion. Initially, a high-dose myeloablative regimen based upon total body irradiation or busulfan regimens was done, probably by previous experiences in treating neoplasm, but promptly, this regime was switched to a myeloablative intermediate regimen based upon a combination of BCNU, etoposide, cytosine arabinoside, and melphalan (BEAM). Ex vivo T cell depletion was substituted by in vivo T cell depletion with ATG without graft manipulation [7]. After the first experiences with high-dose myeloablative with purging CD34+, a recommendation from the EBMT to use intermediate regime was done [16]. In fact, we decided to use an intermediate regimen, due to the initial toxicity reported with high-dose regimens and the absence of benefits in the progression of patients [4]. More recently, a new approach using immunosuppressive drugs such as alemtuzumab to avoid the myeloablative effect has been suggested. The rationale of non-myeloablative HSCT in MS is to suppress relapses acting prior to the onset of irreversible progressive axonal degeneration, to prevent inflammation, and to reduce toxicity in older patient [17, 18].

An important issue of our study is the continued worsening of disability in SPMS patients, despite there were no significant differences in baseline characteristics with RRMS patients according to demographic features, previous relapses, and MS duration previous to AHSCT (in fact, the only difference was the EDSS, but not the increase in EDSS in the previous 2 years).

These results emphasize the necessity of an accurate assessment of the reasons leading to increase in disability. Thus, in RRMS, disability increase was mainly due to relapses or their sequels, whereas in SPMS, the increase in EDSS was due to a sustained progression of disability.

In SPMS patients, all but two worsened in the long term, in line with the results from other series in which 78% of patients progressed after 7-year follow-up [19]. By contrast, no patients with a RRMS form progressed, which is in line with other studies in this clinical form, and RRMS patients respond despite high EDSS, when it was the results of a bout [20, 21].

Burman et al. reported his experience in 34 RRMS patients followed by a mean time of 47.4 months the EDSS improved at 2 years after AHSCT [21]. Klasulova et al., who used a similar AHSCT protocol in 26 MS patients (11 RRMS and 15 SPMS), with a median follow-up of 5 years, reported 100% of RRMS patients remained progression free (median follow-up 19 months for RRMS); meanwhile, all SPMS patients progressed [22].

Mancardi et al. reported 74 MS patients treated with our same AHSCT protocol and followed up for a mean of 48 months, with 71% percentage of the 25 RRMS patients remaining progression free [23]. Compared to our study, their higher baseline EDSS (6.3 vs 5.0), and their higher EDSS the year before AHSCT (4.8 vs 3.7) [23], may explain our higher progression-free rates. This is in agreement with other studies that show that baseline EDSS and specially EDSS in the year before influence response to AHSCT [24] and with our multivariate Cox regression analysis, in which the only predictor factor, was the EDSS in the year before. In fact, in our series, patients with an EDSS equal or lower than 4.0 did not progress in the EDSS (OR 4.9, range 1.1–21.0, *p* = 0.02).

A possible explanation for this finding is that patients with an EDSS higher than 4.0 may actually be already on SPMS phase, as AHSCT has shown a lesser effect on SPMS (where a short-time EDSS improvement followed by posterior worsening has been described) and as SPMS diagnosis is many time difficult at initial stages [25].

Recently, two other papers have reported the experience of AHSCT in RRMS. Nash published a pilot trial with 25 RRMS followed for a mean of 3 years, in which 90.9% reached the progression survival free status, with AHSCT with CD34+ selection, which is different to our procedure [10]. In this work, safety was slightly worse with nearly 100% of patients with degree 4 in the WHO classification secondary effects; meanwhile, in our study, only 10% of patients reached this degree of toxicity. Shevchenko communicated the results of mini-BEAM conditioning regimen in 43 RRMS patients followed by a mean of 48 months. In this series, 86.8% remained progression-free survival, and the procedure was well tolerated [26].

Two other studies with non-myeloablative regimes have been published [17, 18]. In the series reported by Burt et al. [17], in which the main endpoint was the improvement in the EDSS, of the 27 RRMS patients followed for 5 years, 52% of patients improved their EDSS; in 33% patients, the EDSS did not change; and in 14.8%, the EDSS worsened by 1.0 point. Our results are better than those reported by Burt et al.’s study with a 60% of SRD after 6 years of follow-up, despite the conditioning regime used in their trial was based on cyclophosphamide 50 mg/kg/day, 5 to 20 days before stem cell infusion plus either 20 mg of alemtuzumab given 2 days before stem cell infusion to lymphoablation, in order to avoid the deep myeloablation with other regimes; the authors outlined an increase in the EDSS in SPMS and a global worsening at 2 years in 11% out 82 patients. Finally, in the work of Curro et al. with 7 RRMS patients treated with a low intensity lympho-ablative regimen, more than 50% worsened at 5 years [18].

The concept of SRD was initially introduced by Phillips et al. [27] in the post hoc analysis of the efficacy of natalizumab in the Affirm [28] and Sentinel [29] trials, showing that 36% of patients treated with natalizumab achieved this status after 2 years of treatment. More recently, also the CAREMS-I [30] and CAREMS-II [31] trials comparing alemtuzumab with high-dose subcutaneous interferon beta-1a have used the SRD as a tertiary endpoint [32]. Kaplan-Meier estimation to SRD indicated that 52% of alemtuzumab-treated patients achieved SRD after a follow-up of 36 months.

A SRD of 81% with a follow-up of 37 months has been reported in RRMS treated with non-myeloablative autologous hematopoietic stem cells transplantation (AHSCT), in an extension of this study 27 patients that reached 5 years after non-myeloablative AHSCT; 51% were SRD but 15% progressed in disability [11, 17].

Our study has some limitations. First, improvement in function in patients with MS may be in part a consequence of continuing recovery from earlier relapses. However, to our knowledge, no other therapy for relapsing-remitting MS has demonstrated significant and sustained improvement in disability for 4 years (time to first treatment after AHSCT). And second, the absence of a control arm that precludes making direct comparison. Although general conclusions about the real effect of AHSCT in SPMS cannot be obtained, we consider the results obtained in RRMS could be generalized, because the different reports in cohorts of RRMS patients have similar results and are superior to any other therapeutic procedure performed in RRMS.

Our study included 22 RRMS patients followed by a median time of 6 years, and 9 secondary progressive MS (SPMS) followed a mean time of 9 years. This is the longest study with BEAM regimen in RRMS and shows that aggressive RRMS patients have a sustained improvement in disability (60%). Moreover, this protocol was safe, with no deaths related to the AHSCT, and only 10% of severe (grade 4 in the World Health Organization toxicity scale) adverse events.

A baseline pre-AHSCT EDSS ≥3.5 and developing disability progression in the absence of clinical relapses (SPMS) in the previous year implied a poorer response to AHSCT, similar to other studies with high active drugs like natalizumab, alemtuzumab, or even other reports from AHSCT series.

Our work has future implications in two ways: first, we consider that a main endpoint in future trials should be the percentage of patients achieving SRD status and second, criteria to treat MS with highly active immunosuppressant procedures should include only patients in the RRMS phase, since risk benefit in the SPMS is unsure.

In conclusion, to our knowledge, this study is the longest series of RRMS published with the BEAM-ATG regimen followed by AHSCT. We consider this intermediate regimen is safe and very useful to treat RRMS patients that have failed to conventional therapies. Unfortunately, this AHSCT protocol seems to be unable to arrest the progression in SPMS patients. Finally, the importance of accurate evaluation of disability in MS patients must be highlighted, given that patients with fixed disability higher than 4.0, in the previous year to transplant (probably already on the SP phase), have a fivefold higher failure rate than patients with lower disability.
